# Supplementary material for: Are carbon emissions trading and green financial instruments synergistic? -Comprehensive quantitative research based on content analysis
Source: PLoS One. 2024 Mar 7;19(3):e0298601. doi: 10.1371/journal.pone.0298601 (PMC10919601; doi:10.1371/journal.pone.0298601)
Supplement: S1 Data — (ZIP) [file pone.0298601.s001.zip › Data/地级市Z_F工作报告.zip!/╝¬┴╓/╦─╞╜╩╨/└┤╫╘╣1⁄2╓┌║┼í╛┬φ┐╦╩2╛▌═°í┐_2011.docx]

各位代表：

　　现在，我代表四平市人民政府，向大会报告工作，请予审议。

　　一、2010年工作回顾

　　2010年,在省委、省政府和市委的领导下，全市上下认真贯彻落实科学发展观，攻坚克难，求富图强，经济社会呈现出增速加快、活力增强、民生改善、社会和谐的喜人形势，较好地完成了《政府工作报告》确定的各项任务，“十一五”规划目标全面实现。

　　国民经济保持持续健康发展态势。预计生产总值完成810亿元，增长19 %，其中，三次产业增加值分别增长9.4%、27.8%和18.2%。全市一般预算全口径财政收入实现56亿元，增长30.2%，地方级财政收入实现27.9亿元，增长30.7%，其中，市本级一般预算全口径财政收入29亿元，增长30.3%，地方级财政收入12亿元，增长43.4%，财力状况实现较大改善；全市一般预算财政支出完成113.8亿元，增长25.3%。居民消费价格涨幅控制在4.5%以下。

　　——投资力度继续加大，消费拉动稳步增长。预计全社会固定资产投资完成660亿元，同比增长52.7%，全市3000万元以上在建项目达到650个，亿元以上项目180个。大项目、工业项目、税源型项目、域外投资项目、布局合理项目明显增多。加大消费对经济增长拉动作用，预计全市社会消费品零售总额实现290亿元，同比增长18%。热点消费持续升温，全市新落籍汽车2.3万辆；销售家电产品18万多台（部）。全市外贸进出口总额完成2.51亿美元，同比增长71.3%。全年新增个体工商户1.34万户，新创办企业1338户，民营经济主营业务收入、上缴税金大幅提升。全面加强税收征管，营造诚信纳税环境，为纳税人服务、“向纳税人致敬”意识不断增强。

　　——工业经济提质增效，支撑作用不断增强。预计实现工业总产值1100亿元，增加值294亿元，利润36亿元，同比分别增长42%、26%和36.4%。特色产业集聚发展，全国首家换热器公共技术研发中心正式运行，国检中心即将投入使用，为产业发展提供了一流环境和条件；专用车工业园区纳入省规划管理园区；汽车零部件产业集群进一步壮大，生产企业发展到158家；光伏产业异军突起，太阳能电池硅片年生产能力达到1亿片。烟厂扩能改造项目实现了四平人的梦想。传统产业改造升级步伐加快，实施技术改造项目129项，完成投资157亿元，120种新产品试产、投产。节能减排成效明显，万元工业增加值综合能耗降低率达12.8%，降幅居全省第1位。质量和品牌争创工作取得新突破，我市被评为全国“质量兴市”先进市，新增省名牌20个、省著名商标23件、中国驰名商标2件。

　　——农业农村形势喜人，县域经济加快发展。农业农村各项工作取得新进展，粮食产量达到150亿斤以上，创历史最好水平，四个县（市）全部进入全国产粮百强县行列，公主岭市并列成为全国第一产粮大县（市）。全年建设万亩粮食高产示范区40个，新建续建水利工程52项，新增农机动力13.5万千瓦。启动实施“四绿工程”，绿化造林15.2万亩，通过省十年绿化美化吉林大地验收，连续30年无重大森林火灾。“两区一建”成效显著，全市新建扩建牧业小区550个，新增棚膜小区面积1.2万亩，实施“百村示范、千村提升”工程，新农村建设加快推进。农业产业化水平稳步提高，预计农产品加工业产值完成220亿元，同比增长21.5%。新型农村经济合作组织超过2000个，全省4家农村资金互助社全部落户我市。农村“三资”清理工作基本完成，所有乡镇全部建立“三资”代理服务中心。

　　——城市建设稳步提升，社会事业繁荣进步。高起点、高标准完成新一轮城市总体规划和土地利用总体规划修编任务，市区规划面积从51.6平方公里扩容到121平方公里，邀请国内外6所规划设计单位组织编制专项规划。民生建设资金有效增加，投入6950万元完成北河路、邮电街、爱民街等12条市区群众极为关注的市政道路、桥梁翻建维修工程；紫气大路、开运街及东丰路工程开始铺设地下管线；高铁客运站正在紧张施工。全市公路建设完成投资4.7亿元，竣工里程1178公里。四平军民两用机场前期可研工作顺利进行。科技、教育、文化、卫生、体育等社会公共服务能力增强。年内申报国家、省级科技项目131项，14项科技成果获省科技进步奖，四平市国家可持续发展实验区通过专家组考评和国家相关部委联席评审。“国家双拥模范城”六连冠创建工作受到省检查组一致好评。关心下一代工作荣获殊荣。扎实推进“平安四平”建设，人防、物防、技防格局基本形成，设立城市报警、监控中心25个，视频监控点1.5万个，社会治安综合治理不断加强，命案破案率达93.5%。安全生产形势保持稳定，全年无重大以上事故发生。抗洪救灾取得全面胜利。国税、地税、铁路、电力、金融、保险、通讯、石油、烟草等中省直单位为全市经济社会发展作出了积极贡献。

　　——民生工作扎实推进，发展成果惠及于民。坚持把增加就业放在民生工作首位，全年新增城镇就业5.76万人，新增大学生就业5700人，3.7万名下岗失业人员实现再就业，政府出资安排公益岗位1.01万人，城镇登记失业率控制在4%以内。职工和城镇居民医疗保险人数分别达到53.2万人和75.9万人，我市成为全省首个城市及城郊农民社保全覆盖的中心城市。新农合参合率达95.5%。实施“工资倍增计划”，从10月份起，财政供养人员津补贴标准人均月提高600元；全市最低工资标准提高到730元。预计城市居民人均可支配收入16458元，农民人均纯收入6100元，分别增长8.9%和12.6%。棚户区改造、廉租住房建设成效明显。“暖房子”工程完成投资18.7亿元。积极实施“社区行动计划”，市内76个社区落实部门和领导包保责任。

　　——对外开放深入推进，行政改革探索创新。加大开放力度，注重实效，扩大范围，建立广泛联系，组团赴长春、进沈阳、走江西、访湖南、学山东、看铁岭，全年引进省外项目308个，预计完成省外招商引资285亿元。全年争取各类专项资金数额大幅提升，市财政兑付7.2亿元，基本化解了多年拖欠部门、部分企业专项资金占用问题，增强了发展动力。强力推进“资本运作年”，努力构建金融资本汇集洼地。各金融机构新增贷款54.5亿元；担保公司发展到11家，担保额度14亿元；城投债券发行审批顺利；巨元瀚洋换热器转入纳斯达克主板挂牌交易，成为我市第一家上市公司，部分企业上市准备工作加速推进。四平声音和影响力日益增强，为四平发展凝聚了更多人气，成功举办换热器产业国际研讨会，承办全国北方防汛会议；国外知名管理机构介入四平产业发展战略，欧洲专用车厂商开始关注四平；开发区建设明显加快，红嘴高新技术开发区晋升为国家级经济技术开发区，提升了发展的承载力；省政府专门为扶持我市换热器产业发展出台了21条优惠政策，为产业发展创造了良好的环境。

　　把“抓落实”作为政府建设的主题，深化行政审批制度改革，减政放权128项，制定政府令5项、规范性文件16部，依法规范税费和土地出让金减免缓审批，土地出让金收益同比增收1.4亿元。建立向市委汇报工作制度，定期向人大报告工作，向政协通报情况，自觉接受人大法律监督、工作监督和政协民主监督，认真办理人大代表建议84件、政协委员提案174件，办复率达100%。普法工作深入扎实，党风廉政建设责任制全面落实，行政监察和审计监督进一步加强。严格落实信访工作领导责任制，积极化解矛盾纠纷，群众信访总量同比下降43.3%。

　　这些成绩的取得来之不易，是四平广大干部群众开拓创新、努力奋斗的结果，凝结着全市各个方面的智慧和辛勤汗水。我代表市人民政府，向奋战在全市各条战线上的广大劳动者，向给予政府工作大力支持的人大代表、政协委员、各民主党派、工商联、人民团体和社会各界人士，向关心帮助四平建设的驻平部队、武警官兵、中省直单位及海内外朋友表示崇高的敬意和衷心的感谢！

　　在总结成绩的同时，也必须清醒地看到，我市经济社会发展仍存在一些突出的矛盾和问题。主要表现在，经济总量不大，主导产业实力不强；产品结构不优，市场竞争力差，企业效益不高；现代企业管理意识差，资本流入性弱，扩模再生能力不足；农业基础设施还很薄弱，“三化”统筹能力不强；城市功能还不完善，配套设施亟需加快扩张提升，城市管理还存在薄弱环节；民生事业仍有欠账，财力还十分有限，就业和社会保障压力较大，部分群众生活还很困难；政府自身作风建设和效能建设紧迫，思想不够解放，市场经济意识差，创新发展能力亟待提升；县域经济偏弱，一些县（市）区和开发区缺乏支柱产业和龙头企业，获取科技成果、新兴产业渠道狭窄，适应市场要求的人才亟待充分挖掘和引进。这些矛盾和问题在“十二五”期间和今年的工作中必须认真重视，逐步加以解决。

　　二、关于“十二五”《规划》纲要（草案）的说明

　　各位代表, 十二五《规划》纲要（草案）已经提交大会审议。十二五《规划》纲要是承上启下，指导我市今后五年经济社会发展的纲领性文件，是政府履行职责的重要依据，《规划》纲要确定了未来发展的指导思想、发展目标和工作重点，须经由代表讨论，以便把《规划》纲要制定得更加科学、更加完善。为此，我作如下说明。

　　（一）《规划》纲要编制的基本过程

　　十二五《规划》纲要是在市委领导下研究编制的。市委、市政府从2009年4月启动十二五《规划》纲要编制工作，历时一年零九个月，组织有关部门、研究机构和各方面专家，起草并反复修改，数易其稿。市委五届八次全会后，市政府根据市委《建议》精神，在前期工作基础上，深入研究和编制“十二五”规划。《规划》纲要形成过程中，征求了40多位省市知名专家的意见，人大代表、政协委员、各地、各部门、各民主党派、社会团体及各界人士对规划提出千余条宝贵建议。《规划》纲要（草案）的编制过程，是发扬民主、集思广益、科学决策的过程。

　　（二）《规划》纲要的主要内容

　　《规划》纲要共分五篇二十四章四十四节。第一篇为基础环境，科学定位四平经济社会发展现状，分析、判断、把握“十二五”时期的国内外发展环境。第二篇为发展目标，基于“十一五”目标完成情况，研判未来发展趋势，自加压力，提出了经过不懈努力才能实现的发展目标。第三篇为战略布局，主要内容为做强“一核”，建好“三带”，推进“南接北融”的生产力发展布局。第四篇为构建具有四平特色的现代产业体系。第五篇为实施保障措施。

　　（三）《规划》纲要的指导思想

　　“十二五”时期，将是我市破解经济社会发展深层次矛盾，攻坚克难、求富图强，打好全面振兴翻身仗的关键时期。《规划》纲要确定的指导思想是：深入贯彻落实科学发展观，加快经济发展方式转变，紧紧围绕发展和民生两大中心任务，全面实施“一核三带”富民优先战略，坚持投资拉动、项目带动、创新驱动、全民行动，突出“南接北融”，以“一核三带”为先导区，统筹推进四平特色的工业化、城镇化和农业现代化，不断提高核心城市的凝聚力、竞争力、带动力，加快推进社会事业发展，努力让城乡居民生活得更加美好。

　　（四）《规划》纲要编制的几个特点

　　一是“十二五”《规划》纲要是在全面完成“十一五”目标的条件下，确立的发展战略。“十一五”是四平走出了工业连续十年低速徘徊、财政状况低迷的凹谷，步入健康良性发展轨道的5年。从2011年开始的“十二五”将是四平经济社会发展的重要时期，只要我们继续保持当前良好的发展势头，未来5年四平发展将会越来越好，“十二五”规划正处于这样一个承前启后的阶段。

　　二是“一核三带”思想贯穿《规划》纲要始终。“一核三带”富民优先战略是统领全市当前乃至今后一个时期经济社会发展的总纲，“十二五”《规划》纲要在制定过程中以“一核三带”战略为指导，完全符合省委、省政府和市委要求，符合四平发展阶段和市情，增强了《规划》纲要的操作性、实践性。

　　三是主要经济指标增长幅度较大。到2015年，地区生产总值达到2400亿元，年均增长24%；一般预算全口径财政收入实现200亿元，年均增长30%；地方级财政收入实现100亿元，年均增长30%。确立主要经济指标高幅增长的依据是，“十一五”规划全面完成，为加快发展奠定了基础。国内外形势和发展环境有利，国家支持老工业基地振兴、未来宏观经济政策明确等有利条件。更为重要的是，全市上下抢抓机遇、奋力赶超的愿望十分迫切，“十二五”期间打好全面振兴的翻身仗，需要自我加压、乘势快上，在主要经济指标上实现较快增长。

　　四是着重培育战略性新兴产业成为发展重点。针对高新技术产业一直是我市经济结构中“短板”的实际，《规划》纲要确定培育发展新能源汽车、生物技术、新材料、电子信息、节能环保等战略性新兴产业，优化发展结构。

　　三、2011年工作任务

　　2011年是全面实施“十二五”规划的开局之年，也是四平加快转变、加快赶超、加快隆起的关键一年。做好今年工作，要认真谋划开篇，搞好布局，打好基础，合理增速，在关键环节上力求突破、赢得主动，使全局扩张有更大成功。

　　全年主要预期目标是：完成生产总值1010亿元，增长25%；一般预算全口径财政收入70亿元，增长25%；全社会固定资产投资620亿元，增长30%（按新统计口径）；社会消费品零售总额实现339亿元，增长17%；城镇居民人均可支配收入和农民人均纯收入分别实现17775元和6710元，增长8%和10%；居民消费价格涨幅控制在4.8%以内；城镇登记失业率控制在3.75%以内；人口自然增长率控制在4‰以内；万元GDP能耗下降4%。为实现上述目标，重点做好以下工作。

　　（一）全力落实“一核三带”发展战略，为新一轮发展构建新格局

　　“一核三带”生产力战略布局，是四平打好全面振兴翻身仗的关键所在，未来90%的经济总量、80%的人口将汇集于此。 “十二五”时期乃至更长一点时间，做强“一核三带”，特别要从今年开始，凝心聚力做好以下工作：一是深度规划。邀请国内外知名规划设计单位，充分利用区位、资源、土地、劳动力等优势，从资金、技术、投资、消费、人才、物资、产业上合理布局，确定新区定位，打造全新形象、全新功能和全新的增长极，在城区构架、交通组织、建筑、能源、自然景观系统、工运工程做深度的控制性详细规划，完善土地利用规划。二是充分挖掘各种潜力。建立投融资体系，利用国家实施积极财政政策和稳健的货币政策，赴省进京，争取专项、争取国债、争取国际金融信贷支持，挖掘内部潜力，加快资源变资金，资金变资本进程，加大地方前期投入。积极稳妥地推进BT、BOT融资方式，加快基础设施建设，形成良好的投资环境。三是加强区域产业发展研究。对外收集拥有技术、资本、管理、市场优势企业的信息和情报，制定相关政策，采取可行措施，全民行动，攀高结贵，寻求合作伙伴，开发新兴产业。四是培养引进各类专业人才。有计划、有步骤地选送各方面人才，培训、挂职和交流，优先选聘事业发展急需的专业人才，尽快形成推进经济发展专业团队。

　　（二）进一步调整和优化结构，不断提高经济增长质量和效益

　　突出工业主体地位。打造支柱产业集群和重点纳税大户，实现工业总产值1320亿元、增加值345亿元、利润41.4亿元，全年新增产值10亿元以上企业20家，5亿元以上企业30家。加快换热器产业基地建设，形成发展龙头，带动产业集聚。完善四平专用车生产销售基地，吸引鼓励以专用车为主体的汽车厂家来平投资建厂。抓住一汽集团提产扩能的机遇，加快建设四平汽车零部件生产集散地，打造千亿元产业基地。推动医药、食品产业调整产品结构，促使产业向高端、精品、集群配套方向转变。充分挖掘四平老工业基地潜力，形成新的经济增长极。充分发挥开发区（工业园区）先导作用，调整完善规划，加速基础设施建设，落实招大商、招规模企业目标任务，支持开发区在运行机制、选商政策、拓展服务、吸纳人才上创新途径和办法，保持开发区经济总量高速扩张，增速不低于40%。

　　全力推进项目建设。围绕扩大增量，一切资源向项目建设倾斜，工作重心向项目建设转移，构建以工业、农林水利、矿产勘探、交通基础设施、城市建设、现代服务业、社会事业、生态环保八个类别。以开发区、市区、县域三个板块为落脚点的项目支撑体系。

　　注重项目生成。重点开展“地质勘探年”活动，加大油气、地热、煤炭、矽砂、银粉、钠基膨润土等矿产资源勘探与深度开发。市级项目库储备亿元以上项目800个，其中50个5亿元、15个10亿元以上项目达到建议书深度，力争列入省重点项目库。积极争取将我市列入国家支持老工业基地振兴二线城市试点。

　　着力提高企业核心竞争力。年内建设3000万元技改项目100项，完成技改投资200亿元。全年首次投产新产品130种，完成新产品产值90亿元。着力推进全民行动，鼓励支持民营经济和中小企业发展，全年净增个体工商户6000户、私营企业1000家以上。在全市倡导“企业价值源于社会”理念，提高企业管理水平、按章纳税、回报社会的能力和意识。

　　切实抓好招商引资。发挥企业主体和政府主导作用，对世界500强、国内100强和央企紧盯不放，叩门招商，年内力争有2-3户大企业落户四平。全年完成省外招商引资到位资金370亿元，同比增长30%。积极优化出口结构，全年实现进出口总额3亿美元，同比增长20%。

　　（三）巩固农业基础地位，加快推进农业现代化

　　以“三化”统筹，率先实现农业现代化为目标，推进“三区一建”，实现农村发展新跨越。巩固建设好40个粮食高产示范区，全面推进增产30亿斤粮食工程。扎实推进新农村建设，在推进农村经济发展的同时改善人居环境，突出“一核三带”沿线、沿边和环城周边村屯环境整治，彻底解决“城中村”脏乱差问题，15个省级生态乡镇和生态村，118个省级示范村全部达到标准。借助国家加大水利投资政策，倾力抓好灌区续建配套和节水改造、中小河流综合治理、病险水库除险加固、抗旱水源等重点工程。高标准提高城乡绿化水平，推进生态林、生态区建设，以“四绿工程”和“三北”防护林五期工程为重点，完成造林面积20万亩。统筹城乡发展规划，培育一批经济活跃、功能齐全、具有较强承载力和辐射力的中心镇，加快推进城乡一体化发展。

　　（四）以“活力四平”为目标，加快推进新型服务业发展

　　提升发展层次。把目标定位在新型产品东北区位的集散地、国储中心、央企东北仓储中心的目标上，争取辐射力强、更多的商贸名品名店进驻四平。对高铁客运站周边的新商业中心、紫气大路两侧区域，编制好控制性详规，建设聚集专业卖场、商务办公、会展、餐饮、休闲娱乐等商业业态的城市综合体项目。依托东北几何中心的区位优势，结合自身产业特征筹建物流中心、会展中心，加快推进东北亚物流港、北方葡萄酒展示交易集散中心前期工作。发展服务外包、信息产业等生产性服务业；抓好具有四平特色的文化创意、影视、动漫制作等新兴服务业；继续推进万村千乡市场工程，促进农村消费。强化金融资本运作，加快企业上市步伐，由人民银行、银监局牵头，邀请各类金融机构参与县（市）区、开发区项目前期工作。提升旅游品质，围绕“一湖两区”建设，争创全国风景名胜区。加快提升我市房地产业发展，以市场为导向，形成梯次房地产价位。

　　（五）以提高城市承载能力为基础，为城市发展和居民生活提供必备条件

　　完善城市规划，按照新版城市总体规划要求，编制好功能分区及城区内各开发区、工业集中区规划，尽快编制城区绿化系统、水系统、道路交通等专项规划。加快城市基础设施建设，改造维修中央路、铁东七经街等工程24项；完成紫气大路、开运街、兴红街立交桥、东丰路等4项道路桥梁续建工程；实施仁兴街立交桥、纺织路等新建道路桥梁工程13项。强化交通管理，增加环岛、路边停车场等交通设施，打造市区快速路。加强城市管理经营，理顺城市管理体制机制，严格执行土地招拍挂制度，提高城市土地价值和利用效率；规范公共资源经营，城市基础设施项目的资金来源要和土地收益挂钩，封闭运行。广泛运用直接融资、间接融资筹措资金。加快推进“南接北融”，推进交通网络建设，实现快通快联，融入“长、沈”两市一小时经济圈，努力创造长吉平一体发展格局。推进四平支线机场立项工作。

　　（六）切实关注和改善民生，为城乡居民美好生活创造条件

　　以提高城乡居民幸福指数为目标，办好12件民生实事：一是深入推进“社区行动计划”，激活社区功能，畅通民意渠道，按群众需求开展服务，着手解决弃管小区问题，市内76个社区办公场所全部实现宽带、有线全网接入，社区工作人员收入标准再次上涨20%。二是推进就业能力提升，全年新增就业4.8万人，下岗再就业3万人，公益岗位增加20%，农村劳动力转移就业70万人。三是实施安居保障工程，完成棚户区改造拆迁面积60万平方米，新开工200万平方米；公租房、廉租房完成12万平方米，解决2000户城市中低收入家庭的居住困难问题。改造农村泥草房8000户。四是提高城市污水处理能力，全力推进市污水处理厂升级改造扩建，污水处理能力达到11.5万吨/日。五是继续实施暖房子工程，增加供热能力500万平方米，改造管网18公里，既有建筑节能及供热计量改造30万平方米，完善供热信息监管平台和特困群众供热保障机制。六是实施燃气改造工程，新建、改建天然气加气站4座、管网4万延长米，日供气量增加5000立方米。七是加快城乡路网建设，着手启动城市快速路工程，抓好过境高速公路、国道工程建设，建设农村公路1500公里，基本实现全市行政村100%通水泥（沥青）路。八是全面搞好绿化美化工程，重点建设市区西南侧森林绿带，抓好市区出口和街路广场绿化，推进社区、广场、机关和小区庭院绿化，打造绿意盎然的城市。九是提高保障水平，建立帮贫扶困专项基金，解决困难群众基本生活；提升低保水平，城市低保金月提至252元，增长18%；农村低保年人均增至1360元，增长13%。十是基本解决中小学校舍安全问题，维修加固中小学校舍58万平方米，重建中小学校舍40万平方米。十一是实施文化惠民工程，扶持建设52个乡镇综合文化站，69个农村文化大院。十二是实施农村饮水安全工程，建设饮水安全工程200处，解决10万农村人口饮水安全问题。

　　（七）加快社会事业发展，打造文明进步的和谐四平

　　教育着力解决公平问题，大力发展学前教育，探索职业教育集团化办学模式。卫生着力解决信誉问题，在全市开展“10所优质医院、20家放心药店、100名满意医生”评选活动。文化着力解决活力问题，促进文化与经济发展相融合。广电着力解决创新问题，创新广播电视节目形式，增设法制、经济类栏目，宣传发达地区经济理念、先进经验。体育着力解决活跃问题，广泛开展全民健身活动，争取承办特色赛事，扩大四平传播领域。计生着力解决质量问题，深化生育关怀，稳定低生育水平。贯彻民族区域自治法，高度重视民族宗教工作，维护民族团结。扎实开展双拥共建活动，巩固全国双拥模范城成果，加强国防后备力量建设，走融合发展之路。加强人防工程的开发利用。搞好第六次人口普查，抓好防洪抗旱、森林防火、气象和地震防灾减灾等工作。加快发展邮政、通信事业，更好地服务于经济社会发展。

　　全力维护社会稳定。进一步强化信访责任落实，提高工作的预见性和实效性，集中力量解决涉及民生的信访问题，积极预防和妥善处置社会矛盾和群体性事件。不断深化法制宣传教育，开展打击违法使用土地专项整治工作。社会治安综合治理着力解决“群众安心”问题，保持对刑事犯罪的高压严打态势，切实提高人民群众的安全感。突出预防为主，抓好重点行业和重点部位公共安全专项整治，确保不发生重特大安全生产事故。整顿和规范市场秩序，及时查处串通涨价、哄抬物价等行为，确保消费者利益。加强食品药品监管，建立食品安全综合协调机制，保障人民群众身体健康和生命安全。健全应急预警体系，提高处置突发公共事件能力，努力营造和谐稳定的社会环境。

　　各位代表：

　　全面完成今年和“十二五”期间的任务，对各级政府提出了更高的要求，必须大力加强政府自身改革和建设。

　　建设创新经济型政府，做到“懂经济、会算账”。把行政行为与市场行为一致起来，在公共产品提供、招商引资上项目、企业培育、城市建设过程中，严格进行成本核算，按经济规律配置资源、评判项目，以最合理的投入为人民办更多实事，建设懂经济、会落实、能干事、守护公共利益的公务员队伍。鼓励创新、善待挫折、激励成功，营造干事创业、勇于创新的浓厚氛围。继续提高财政供养人员津补贴标准，下半年争取达到全省平均水平。

　　建设优质服务型政府，做到“讲服务、有笑脸”。哪里有需要，就服务到哪里。要用硬措施来整治软环境，加大问责追究力度，严查严办涉软案件。创新服务方式，多设路标，少设路障，对办事流程一次性告知，一站式服务，一次性办结，努力使四平成为环境优、成本低、回报高、适应时代与发展要求的创业城市。

　　建设规范效能型政府，做到“谋发展、说行话”。规范和理顺政府行政运行机制，合理调配人员编制。政府各部门要各负其责，协调配合，服务中心和大局，做到政令畅通，合力攻坚。全体公务人员要提高业务素质，发挥能动作用，主动加压、埋头苦干，“不议论、不争论，用实践作结论”，把工作中心放到推动发展的重要任务上来，放到加快建设的重点工程上来，放到改善民生的重大课题上来，摒弃“各自为政、互相推诿”的麻木作风，形成千军万马一条心，众人划桨开大船的良好氛围。

　　建设法治廉洁型政府，做到“用公权、守规矩”。坚持在市委的领导下开展工作，认真执行人大及其常委会决议，自觉接受人大法律监督、工作监督和政协民主监督，积极办理人大代表建议、政协委员提案。强化政府法制监督，加快推进阳光政务，全面公开经济社会发展重大事项、行政审批项目、重大财政支出和公共资源使用情况，公开事关民生的人大代表建议、政协委员提案办理情况。全面落实党风廉政建设责任制，推进惩治和预防腐败体系建设，加强行政监察、审计监督，强化公共权力规范运行，树立勤政高效、清正廉洁的政府形象。

　　各位代表，回首过去历程，成就鼓舞人心；展望发展前景，目标催人奋进。让我们在市委的坚强领导下，攻坚克难、求富图强，为实现“十二五”规划目标，为城乡居民生活得更加美好而努力奋斗！
